# No effect of a widespread pharmaceutical pollutant on behavior, physiology, or morphology in guppies

**DOI:** 10.1093/beheco/arag080

**Published:** 2026-07-14

**Authors:** Kate N Fergusson, Stephanie L Hannington, Jack A Brand, James L Tanner, Amanda K Pettersen, Celine T Goulet, Josefin Sundin, Minna Saaristo, Michael G Bertram, Bob B M Wong, Jake M Martin

**Affiliations:** School of Biological Sciences, Monash University, 25 Rainforest Walk, Clayton, Victoria 3800, Australia; School of Biological Sciences, Monash University, 25 Rainforest Walk, Clayton, Victoria 3800, Australia; School of Biological Sciences, Monash University, 25 Rainforest Walk, Clayton, Victoria 3800, Australia; Institute of Zoology, Zoological Society of London, Outer Circle, Regent’s Park, London NW1 4RY, United Kingdom; Department of Wildlife, Fish, and Environmental Studies, Swedish University of Agricultural Sciences, Skogsmarksgränd 17, Umeå 907 36, Sweden; School of Biological Sciences, Monash University, 25 Rainforest Walk, Clayton, Victoria 3800, Australia; School of Biological Sciences, Monash University, 25 Rainforest Walk, Clayton, Victoria 3800, Australia; School of Life and Environmental Sciences, The University of Sydney, Science Road, Camperdown, New South Wales 2050, Australia; School of Biological Sciences, Monash University, 25 Rainforest Walk, Clayton, Victoria 3800, Australia; Department of Aquatic Resources, Swedish University of Agricultural Sciences, Stångholmsvägen 2, SE-17893, Drottningholm, Sweden; School of Biological Sciences, Monash University, 25 Rainforest Walk, Clayton, Victoria 3800, Australia; Environment Protection Authority Victoria, EPA Science, Ernest Jones Drive, Macleod, Victoria 3085, Australia; School of Biological Sciences, Monash University, 25 Rainforest Walk, Clayton, Victoria 3800, Australia; Department of Wildlife, Fish, and Environmental Studies, Swedish University of Agricultural Sciences, Skogsmarksgränd 17, Umeå 907 36, Sweden; Department of Zoology, Stockholm University, Svante Arrhenius väg 18b, Stockholm 114 18, Sweden; School of Biological Sciences, Monash University, 25 Rainforest Walk, Clayton, Victoria 3800, Australia; School of Biological Sciences, Monash University, 25 Rainforest Walk, Clayton, Victoria 3800, Australia; Department of Wildlife, Fish, and Environmental Studies, Swedish University of Agricultural Sciences, Skogsmarksgränd 17, Umeå 907 36, Sweden; School of Life and Environmental Sciences, Deakin University, 75 Pigdons Road, Waurn Ponds, Victoria 3216, Australia

**Keywords:** antidepressants, fluoxetine, foraging, metabolic rate, multigenerational, predator

## Abstract

Pharmaceutical pollution is a global environmental threat, with antidepressants contributing a substantial proportion of pharmaceutical contaminant detections worldwide. The antidepressant fluoxetine is commonly detected in aquatic environments and has been reported to impact ecologically important behavioral and physiological traits in exposed wildlife. However, relatively few studies have examined possible effects of fluoxetine on both behavior and metabolic rate, particularly over ecologically relevant exposure timescales. Accordingly, we investigated the impacts of long-term fluoxetine exposure on foraging and antipredator behavior, metabolic rate, and morphology in female guppies (*Poecilia reticulata*). Fish were exposed to 1 of 3 treatments—an unexposed control (0 ng L^−1^), or low or high fluoxetine (mean measured concentrations: 30 ng L^−1^ and 292 ng L^−1^, respectively)—for 8 mo in a semi-natural mesocosm system, corresponding to ∼2 to 3 overlapping generations. Following exposure, we quantified foraging and antipredator behavior in the presence or absence of a live predator, the spangled perch (*Leiopotherapon unicolor*). We then measured the rate of oxygen consumption ($\dot{V}$O_2_) of the guppies as a proxy for standard metabolic rate, and recorded mass, standard length, and body condition as morphological measurements. Our results show that long-term fluoxetine exposure did not significantly affect the behavior, metabolic rate, or morphology of female guppies. Considered alongside previous work, our findings emphasize the importance of exposure duration in mediating the impacts of fluoxetine on fitness-related traits, with continued research being essential in identifying potential mechanisms that underpin these findings.

## Teaser

Pharmaceutical pollution poses a growing threat to aquatic wildlife. Eight-month exposure to the antidepressant fluoxetine did not impact behavior, physiology, or morphology in female guppies, despite previous studies detecting impacts following short-term exposure. Our study highlights the importance of considering exposure duration in mediating the effects of fluoxetine on fitness-related traits.

## Introduction

Contamination of aquatic environments by pharmaceuticals is a major ecological threat. With thousands of pharmaceuticals commercially available, over 990 pharmaceutical compounds (or their transformation products) have been detected in aquatic ecosystems globally (Patel et al. 2019; Graumnitz and Jungmann 2021). Antidepressants are a particularly concerning group of pharmaceutical pollutants due to their pervasive use as psychotherapeutics and their ubiquity in the environment (Wilkinson et al. 2022). Like many other pharmaceuticals, antidepressants typically enter the environment following use and excretion while they are still in a bioactive form (Wang et al. 2021; Brodin et al. 2024). As current wastewater treatment processes are generally inadequate in removing pharmaceutical residues (Baresel et al. 2019; Saaristo et al. 2023), sewage effluent is a primary source of environmental contamination (Wronski and Brooks 2023).

The selective serotonin reuptake inhibitor (SSRI) fluoxetine is one of the most common antidepressant contaminants, with environmental concentrations typically ranging between <1 and 330 ng L^−1^ in freshwater systems (Mole and Brooks 2019; Gould et al. 2021; Sumpter and Margiotta-Casaluci 2022). SSRIs elicit their therapeutic effects by inhibiting the reuptake of serotonin, which increases extracellular serotonin levels in the brain (McDonald 2017; Gould et al. 2021). As the primary target receptor of fluoxetine (the serotonin transporter)—and the serotonergic system more generally—is evolutionarily conserved across a range of animal taxa (Gunnarsson et al. 2008), fluoxetine has a high potential to impact nontarget organisms. Indeed, fluoxetine has been found to bioaccumulate in the tissues of exposed wildlife (Pan et al. 2018; Richmond et al. 2018; Yan et al. 2020), as well as disrupt a range of ecologically important physiological, morphological, and behavioral traits (Bidel et al. 2016; Thoré et al. 2020; Correia et al. 2023).

Antipredator behavior is a particularly important trait regarding the effects of pharmaceuticals in the environment. In prey species, adaptive behavioral responses are crucial for avoiding predation, as failing to respond to a predator effectively can be fatal (Zanette and Clinchy 2019; Allen et al. 2022). This has led to considerable research investigating the impacts of pharmaceutical contamination on antipredator behavioral traits in wildlife, such as avoidance behaviors (eg Fursdon et al. 2019), fast-start escape responses (eg Jonsson et al. 2019), freezing to avoid detection (eg Martin et al. 2017), and camouflage (eg Ford and Feuerhelm 2020). In a natural setting, time spent engaged in antipredator behaviors must often be traded against other important behaviors, such as foraging (Turney and Godin 2014) and reproduction (discussed in Glavaschi et al. 2022), with prey species needing to adjust their behavior according to the perceived level of risk. However, studies to date exploring the effects of pharmaceutical contaminants, such as fluoxetine, on antipredator behaviors have rarely considered such adjustments with other ecologically important behaviors (but see Peters et al. 2017; Thoré et al. 2019; discussed in Bertram et al. 2022).

Another area in need of further investigation is the potential effects of pharmaceuticals on behavior and physiology in combination, with research particularly lacking on potential effects of fluoxetine on metabolic rate (but see Tan et al. 2020; Fergusson et al. 2024). This is surprising given the role that the serotonergic system plays in regulating various physiological functions, including energy expenditure (McGlashon et al. 2015; Moon et al. 2022). Moreover, metabolism is known to influence all biological activities and is closely linked to behavior (Mathot et al. 2019; Wu and Seebacher 2022). For example, it has been reported that European sea bass (*Dicentrarchus labrax*) with higher metabolic rates take more risks during foraging compared with conspecifics with lower metabolic rates (Killen et al. 2011). Given these interconnections, examining how contaminants like fluoxetine affect metabolic rate and behavior in combination could therefore provide critical insights into the broader ecological consequences of pharmaceutical pollution.

In addition, it is important in pharmaceutical ecotoxicology research to consider exposure duration. Many drugs can persist in the environment for long periods of time because they are resistant to environmental degradation (O'Flynn et al. 2021; Maculewicz et al. 2022) or are continually replenished from sources such as sewage effluent (ie “pseudo-persistence”; Arnold et al. 2014; Correia et al. 2023). Recent long-term exposure studies have not only uncovered a wide range of fluoxetine-induced effects on fitness-related traits (eg Mason et al. 2021; Polverino et al. 2021; Henry et al. 2022), but also highlighted that such effects may be duration- or generation-dependent (Minguez et al. 2015; Vera-Chang et al. 2018; Heyland et al. 2020; Thoré et al. 2021b; Fergusson et al. 2024). Among these recent studies, Fergusson et al. (2024) examined the effects of long-term fluoxetine exposure on boldness and exploratory behavior, as well as metabolic rate and morphology. Interestingly, no effects of fluoxetine exposure were observed on any of the traits measured except for body condition. Considering there is currently a dearth of research on the long-term impacts of fluoxetine exposure (Martin et al. 2025), and evidence to suggest duration- or generation-dependent effects, there is a clear need for research under extended exposure periods (ie spanning multiple generations).

Here, we capitalize on the same long-term semi-natural mesocosm population system as Fergusson et al. (2024) to investigate whether 8-mo (corresponding to ∼2 to 3 overlapping generations) exposure to environmentally realistic fluoxetine concentrations would alter foraging and antipredator behavior, metabolic rate, and morphology in female guppies (*Poecilia reticulata*), complementing previous work in males. In Fergusson et al. (2024), behavior was examined in the presence of a live spangled perch (*Leiopotherapon unicolor*) in an open arena, whereas in the present study, we introduce a foraging context to assess behavior under risk-reward conditions. If the effects of long-term exposure to fluoxetine would reflect those of acute exposure studies (eg Pelli and Connaughton 2015; Martin et al. 2017), we would generally expect to observe reduced antipredator responses in fluoxetine-exposed guppies, with fish partaking in risky behaviors in the presence of the spangled perch (ie being more active and engaging more in foraging behavior despite the potential threat of predation). Alternatively, if chronic exposure leads to increased habituation or tolerance to the drug (eg as a result of neuroadaptive changes; Targum 2014), it is possible that no differences in antipredator behavior would be observed, as seen following long-term exposure in male guppies (Fergusson et al. 2024). We also measured oxygen consumption as a proxy for standard metabolic rate, hypothesizing that fluoxetine exposure would alter metabolic rate given the known role of the serotonergic system in modulating energy intake and expenditure. While this is not always the case, as demonstrated by the lack of an observed effect in male guppies (Fergusson et al. 2024), changes in metabolic rate have been reported in female guppies following a 15-mo exposure to fluoxetine (Tan et al. 2020). Lastly, we recorded mass, standard length, and body condition (scaled mass index) to determine whether fluoxetine exposure impacted morphology.

## Materials and methods

This study followed the EthoCRED reporting guidelines for behavioral ecotoxicity studies (Bertram et al. 2025). A systematic list of the criteria is provided in File S2 (see Supplementary material).

### Study species

Guppies are a small freshwater fish species native to Trinidad and the north of South America (Rosen and Bailey 1963) that have been introduced to various tropical and sub-tropical regions worldwide (Deacon et al. 2011). The species are also a well-suited model for investigating the ecological effects of fluoxetine exposure. First, in many parts of the world, guppies occur in environments close to human habitation where exposure to pharmaceutical pollutants, including fluoxetine, is likely (Widianarko et al. 2000; Araújo et al. 2009). More generally, the guppy is a commonly used model in behavioral ecology (eg Burns 2008) and ecotoxicology (eg Norrgren 2012; Thoré et al. 2021a). We used adult female guppies in this study because, due to their larger size compared with male conspecifics, they are at a greater risk of predation as they are perceived by predators to be a more profitable choice of prey (Pocklington and Dill 1995). Additionally, female fish are suggested to face a greater trade-off between resource acquisition and predation risk (Britton and Moser 1982; Pärssinen et al. 2021; Aceves-Fonseca et al. 2022) and show higher foraging rates in the wild than males (Heinen et al. 2013). Males, on the other hand, tend to be more active, allocating more time to activities such as searching for, and courting, mates (Heinen et al. 2013).

### Animal collection and housing

#### Guppies

This study utilized a long-term mesocosm population system to investigate the ecological impacts of environmentally relevant fluoxetine exposure on guppies. The founding population was collected in November 2016 from Alligator Creek (19°26′18″S, 146°57′01″E), a rainforest-fed stream located within the Bowling Green Bay National Park in Townsville, Queensland, Australia (collection permit: WITK17685216). The founding population, consisting of 3,600 adults (50:50 sex ratio), were randomly and equally distributed into 12 identical stainless steel mesocosm tanks (648 L; 180 × 60 × 60 cm; water depth: 30 cm) located within a temperature-controlled greenhouse facility at Monash University, Victoria, Australia (Fig. S1). Mesocosm tanks were monitored weekly for temperature (mean = 23.26 °C, SD = 0.91 °C, *n* = 520) and pH (mean = 7.15, SD = 0.68, *n* = 515; see Table S1 for summary statistics of each mesocosm tank) and were kept under a natural light:dark cycle (average 12:12 h light:dark). Each tank contained a 2 cm layer of 7 mm pebble substrate, aquatic plants for refuge (ie Java moss, *Taxiphyllum barbieri*), and carbon-filtered tap water aerated by commercial air pumps (Resun LP100). Fish were fed once per day to apparent satiation with commercial fish food (Aquasonic Nutra Xtreme C1 pellets, 0.8 mm). Exposure to fluoxetine commenced 5 mo after the founding populations were introduced into the mesocosm tanks (see “Chemical exposure and analyses”).

#### Spangled perch

We used adult spangled perch (standard length: 5.53 ± 0.08 cm, *n* = 11) sourced from a commercial supplier (Australian Native Fish Enterprise) as predatory stimuli in behavioral trials (see “Foraging under the perceived threat of predation”), which were originally collected from a wild population in Kallangur, Queensland, Australia. Spangled perch were housed in large, aerated tanks filled with carbon-filtered tap water (60 × 30 × 30 cm; water depth: 20 cm; 2 fish per tank) and were acclimated to laboratory conditions for 2 wk prior to the beginning of behavioral trials. Tanks were maintained at 24 °C under a 12:12 light:dark cycle and perch were fed artificial pellets (Hikari cichlid gold pellets; 3.2 to 3.7 mm) to apparent satiation once daily. The predators were not exposed to fluoxetine. Spangled perch are opportunistic carnivores, with a substantial portion of their diet consisting of smaller fish species (Medeiros and Arthington 2014). Moreover, they have been observed to exist sympatrically with guppies in Australia, including the source population of guppies used in this study (pers. obs.). It has also been demonstrated that guppies are able to differentiate between a spangled perch and a nonpredatory heterospecific (rainbowfish, *Melanotaenia splendida*; Fursdon et al. 2019).

### Chemical exposure and analyses

Five months after the founder guppies were introduced into the mesocosm system, each of the 12 independent mesocosm populations were randomly allocated to 1 of 3 treatments: solvent control (ie unexposed; *n* = 4 mesocosms), low fluoxetine (nominal concentration: 30 ng L^−1^; *n* = 4 mesocosms), or high fluoxetine (nominal concentration: 300 ng L^−1^; *n* = 4 mesocosms). The nominal low-fluoxetine treatment was based on fluoxetine concentrations detected in surface waters, whereas the high-fluoxetine treatment was based on higher ranges of environmental detections (reviewed in Mole and Brooks 2019). Guppies were exposed to these concentrations for 8 mo, which represented ∼2 to 3 overlapping generations (Reznick et al. 1997).

To achieve the nominal exposure concentrations, a static renewal system was used. This involved dosing each mesocosm tank with a fluoxetine stock solution—or, in the case of control tanks, a methanol solvent control (see below)—twice weekly; once directly after a 20% water change and once 3 d after. As fluoxetine was dissolved using methanol (HPLC grade, ≥99.9%), each control tank received 1 mL of methanol mixed into 1 L of reverse osmosis water as a solvent control (ie 2 mL methanol per 648,000 mL water per week; <0.001%). Stock solutions were created by dissolving fluoxetine hydrochloride (≥98% purity; Sigma Aldrich; product number: F132; CAS: 56296-78-7) at the desired concentrations depending on the treatment (low fluoxetine: 10 μg in 1 mL methanol; high fluoxetine: 100 μg in 1 mL methanol) in 1 L of reverse osmosis water.

Two replicate water samples were taken from each of the low- and high-fluoxetine tanks, as well as half of the control tanks (selected at random), every month using a serological pipette. Samples were analyzed using solid phase extraction and liquid chromatography coupled to tandem mass spectrometry (LC-MS) by Envirolab Services (MPL Laboratories; NATA accreditation: 2901; accredited for compliance with ISO/IEC: 17025), with a minimum detection limit of 2 ng L^−1^. A detailed description of this protocol can be found in Bertram et al. (2018).

### Foraging under the perceived threat of predation

Focal females were separated from the main population and isolated within their respective mesocosm tanks in fine-mesh stainless steel cylindrical isolation chambers (diameter × height: 35 cm × 32 cm) 1 wk prior to experimental trials. This was done by catching all females in the mesocosms with nets, and then placing them in the steel cages. Confining females into these isolation chambers facilitated the capture of fish for experiments and reduced the risk of sampling bias (eg inadvertently catching the largest or slowest fish). We avoided females showing obvious signs of gravidity (ie an extended abdomen). After behavioral trials and morphological measurements were complete (see “Morphological measurements”), guppies were returned to their respective mesocosm tanks.

**Figure 1:**
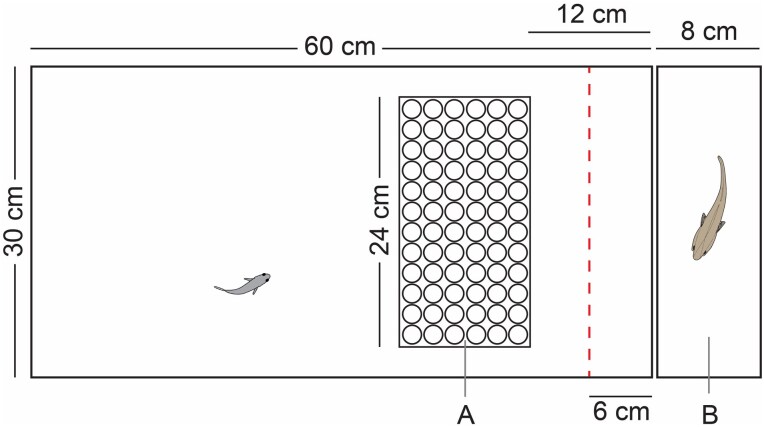
A diagram of the observation tank (left compartment) used to measure the potential impact of fluoxetine exposure on foraging behavior under the perceived threat of predation. The observation tank contained a female guppy and a 72-well foraging plate (a), which was partially covered by sand, and on top of which 20 bloodworms (*Chironomidae* sp.) were randomly placed. The predator compartment (right compartment, b) contained a spangled perch (*L. unicolor*) in the predator treatment and was empty in the no-predator treatment. The dashed line represents the predator strike zone. A photo of the setup is presented in the Fig. S2.

A 3 × 2 independent factorial design was used to test for the potential effects of fluoxetine exposure on foraging behavior under predation risk, where female guppies from each fluoxetine treatment were observed either in the presence or absence of a predatory spangled perch (predator absent: control *n* = 31, low fluoxetine *n* = 28, high fluoxetine *n* = 30; predator present: control *n* = 30, low fluoxetine *n* = 31, high fluoxetine *n* = 31; as described in Fergusson et al. 2024). Guppies have been previously shown to adjust their foraging behavior under predation risk, whereby they reduced their feeding rate when a predator was present compared with when it was absent (Dugatkin and Godin 1992). Behavioral trials were conducted in an observation tank (60 × 30 × 30 cm) that had a separate glass compartment (8 × 30 × 30 cm) on 1 end (hereafter referred to as the “predator compartment”; Figs. 1 and S2). Both the observation tank and the predator compartment were illuminated using 2 fluorescent lights (5,000 K), contained a 2 cm layer of white sand lining the bottom, and were filled with carbon-filtered tap water (water depth: 20 cm) that was maintained between 24 and 25 °C. The external surfaces of the observation tank and predator compartment were frosted to prevent visual disturbance during trials. In the observation tank, there was a designated foraging area that contained a large foraging plate, partially covered with sand, with 72 evenly distributed shallow wells. The predator compartment was placed 12 cm away from the foraging area to ensure that the guppies had to approach the predator compartment to forage and, therefore, increase the risk of predation to consume the food. Behavioral trials were conducted over a 6-d period (8–13 December 2017). To control for any potential side bias (Jeswiet and Godin 2011), we randomized the side of the tank that the foraging area and predator compartment were placed between trials. Prior to each trial, 20 bloodworms (*Chironomidae* sp.) were randomly distributed across the 72 wells (Figs. 1 and S2). Guppies were not fed for 24 h before the behavioral trials to standardize hunger levels and ensure consistency across individuals tested on different experimental days. Observation tanks were drained and refilled between trials to avoid any potential cross-contamination of conspecific chemical cues between trials.

The female guppies were collected from the isolation chambers in the mesocosms using a net and were transported in aerated containers to the nearby laboratory facility where the behavioral experiments were performed. At the beginning of each trial, an individual guppy was randomly allocated to an observation tank and placed in an opaque cylindrical chamber within this tank for a 20-min acclimation period. The chamber was then lifted manually, and the focal fish was able to freely explore and forage within the observation tank for 60 min. All trials were filmed from above (using Panasonic HC-V180 cameras) and their behaviors were later quantified using a behavioral analysis key-logging software (BORIS version 5.1.3; Friard and Gamba 2016). To enable blind analysis of the videos, an ID number tag that did not reveal the treatment history of the guppies was placed in view of the camera on each observation tank. In total, 6 observation tanks were filmed simultaneously. To quantify the propensity to forage, we measured the time taken to first consume a prey item and the number of entries into the foraging area. To quantify antipredator behavior, we measured the time taken for the focal fish to enter a predetermined predator strike zone (s). The “strike zone” refers to a 6 × 30 cm area closest to the predator compartment, which was based on the striking range of the pike cichlid (*Crenicichla alta*)—a similar-sized guppy predator to the spangled perch used in our study (Walker et al. 2005). We also measured swimming activity levels during the trial using Tracker (TrackerV8; Open Source Physics, USA)—a video analysis and modeling tool—whereby mean velocity (cm/s) was calculated using semi-automated tracking (a position every 75 s at 25 frames/s). All videos were scored blind (by S.L.H. and C.T.G.) to fluoxetine treatment to avoid any potential observer bias, by using the aforementioned ID number.

### Standard metabolic rate

To test for the potential effects of fluoxetine exposure on the standard metabolic rate (hereon referred to as “metabolic rate”) of female guppies, we measured the rate of oxygen consumption ($\dot{V}$O_2_) of guppies from each exposure treatment (unexposed: *n* = 35, low fluoxetine: *n* = 34, high fluoxetine: *n* = 36) as described in Fergusson et al. (2024). Focal females were randomly selected from the isolation chambers and transported in aerated containers to the nearby laboratory facility, where metabolic rate was measured. As recent feeding can affect metabolic rate (Jobling 1981), focal fish were not fed for 24 h prior to metabolic rate measurements to standardize hunger and ensure consistency across individuals tested on different experimental days. All trials were conducted in a dark room kept at 24 °C to reduce visual disturbance. Guppies were individually placed inside 100 mL glass vials of sterile pasteurized tap water at 24 °C, with a precalibrated, nonconsumptive oxygen sensor spot on the base of each vial. Oxygen saturation was then measured using a 24-channel PreSens sensor dish reader (Sensor Dish Reader, SDR2, PreSens Precision Sensing, Germany) in 18 vials simultaneously (12 experimental and 6 control vials) at 1-min intervals for 3 h. The first 30 min was excluded from analysis to allow the fish to acclimate (Olito et al. 2017). A total of 4 vials could be fitted simultaneously on 1 reader, with 3 vials containing a single female each and 1 vial containing pasteurized water only to account for any potential background microbial activity. Metabolic rate measurements were conducted over a 3-d period (11–13 January 2018).

The sensor spot determined the rate of oxygen consumption ($\dot{V}$O_2_) from calculating the change of O_2_ saturation (*m*_a_; % h^−1^) as $\dot{V}$O_2_ = − [(*m*_a_ – *m*_c_)/100] × *V* × *β*O_2_ (White et al. 2011), where *m*_c_ is the rate of change of O_2_ saturation for vials containing only sterile aged water, βO_2_ is the oxygen capacitance of the sterile aged water and is the volume of water (ie the volume of water minus the fish mass). We estimated the *m*_a_ and *m*_c_ parameters by ranking local linear regressions (*rankLocReg* function, *LoLinR* package; Olito et al. 2017) to provide a calculation for $\dot{V}$O_2_. Each sensor spot was calibrated with sterile aged water (ie 100% air saturated) and water containing 2% sodium sulfite (0% air saturated). Using the calorific conversion factor (ie 20.08 J mL^−1^ O_2_), $\dot{V}$O_2_ was converted to metabolic rate (mJ h^−1^). This conversion enabled the rate of oxygen consumption to be measured in relation to differently sized females (ie different mass; Lighton 2018). For a detailed list of methodological information following the proposed guidelines of Killen et al. (2021) for reporting methods of aquatic respirometry, see Table S2.

### Morphological measurements

Morphological measurements of guppies were taken following both behavioral and metabolic rate measurements. Standard length (ie from the tip of the snout to the caudal peduncle) was measured using digital calipers (±0.01 mm) and body mass (±0.0001 g) was measured using digital scales (Scientech ZSA-210). To obtain measurements, fish were gently caught with a hand net and placed onto waterproof grid paper and photographed, remaining in the net to ensure they were still for measurements. They were then blotted with absorbent paper and placed into a container of water sitting on top of the balance to measure body mass. Scaled mass index was also calculated because body condition is closely linked to fitness and life-history traits (Karametsidis et al. 2023), as well as behaviors such as foraging and antipredator responses (Siegal et al. 2022).

### Ethical note

The research was conducted in accordance with relevant state and federal laws, and all housing and experimental procedures were approved by the Monash University Animal Ethics Committee (BSCI/2016/06 and BSCI/2017/38).

### Statistical analysis

Data were analyzed using R version 4.2.1 (R Core Team 2022). Data were log transformed where necessary to approximate a Gaussian error distribution, and continuous predictors were scaled (mean = 0, SD = 1) to improve the interpretability of main effects. Global models for the behavioral assay included fluoxetine treatment, predator treatment (predator present or absent), body mass, experimental day (1 to 6), water temperature, and predator side (left or right) as fixed effects. Predator ID was included in initial models; however, as it did not explain any substantial variation in the data, it was excluded from the final analysis. We also included the 2-way interaction between fluoxetine treatment and predator treatment, as well as fluoxetine treatment and body mass. Mass was included as a covariate because it has previously been shown to be associated with various behaviors, such as boldness (Brown et al. 2007; Si et al. 2023) and foraging behavior (Fonds et al. 1992; Byström et al. 2006), in fish. Where the inclusion of covariates did not improve model fit, as tested by Akaike information criterion comparisons, they were removed from the model (see Table S3 for the final models). Given that a high proportion of guppies never ate, we converted the time taken to first consume a prey item into a binary score (1: fish consumed at least 1 prey item; 0: fish did not consume any prey items). We then conducted a generalized linear mixed-effects model (GLMM; *glmer* function, *lme4* package; Bates et al. 2015) with a binomial distribution. For the number of entries into the foraging area, we used a GLMM with a Poisson distribution. Finally, to analyze the time taken to enter the predator strike zone and mean velocity, we used linear mixed-effects models (LME; *lmer* function, *lme4* package; Bates et al. 2015). All models included mesocosm ID as a random effect. Where overdispersion was detected in the GLMMs, fish ID was included as an observation-level random intercept.

To analyze the potential effect of fluoxetine exposure on metabolic rate, we used an LME model with fluoxetine treatment, body mass, the 2-way interaction between fluoxetine treatment and body mass, and time of day (morning or afternoon) as fixed effects, and mesocosm ID as a random effect. We applied a log_10_ transformation to body mass and metabolic rate to satisfy the assumption of homoscedasticity. Linear mixed-effects models were also used to analyze the potential effects of fluoxetine exposure on guppy body mass, standard length, and body condition. We calculated scaled mass index as a proxy for body condition. Specifically, a standard major axis regression was performed on the log of body mass and standard length of fish (*sma* function, *smatr* package; Warton et al. 2012). We then calculated a beta coefficient, which was used to obtain the scaled mass for each fish. Linear mixed-effects models on morphological traits included fluoxetine treatment as a fixed effect and mesocosm ID as a random effect.

## Results

### Chemical analyses

The mean measured exposure concentrations of fluoxetine were 30 ng L^−1^ (SD = 12.84, *n* = 36) for the low treatment and 291.90 ng L^−1^ (SD = 156.59, *n* = 36) for the high treatment. No fluoxetine contamination was detected in the unexposed mesocosm tanks (all under the limit of detection; <2 ng L^−1^, *n* = 18). Refer to Table S4 for raw values.

### Foraging under the perceived threat of predation

**Figure 2:**
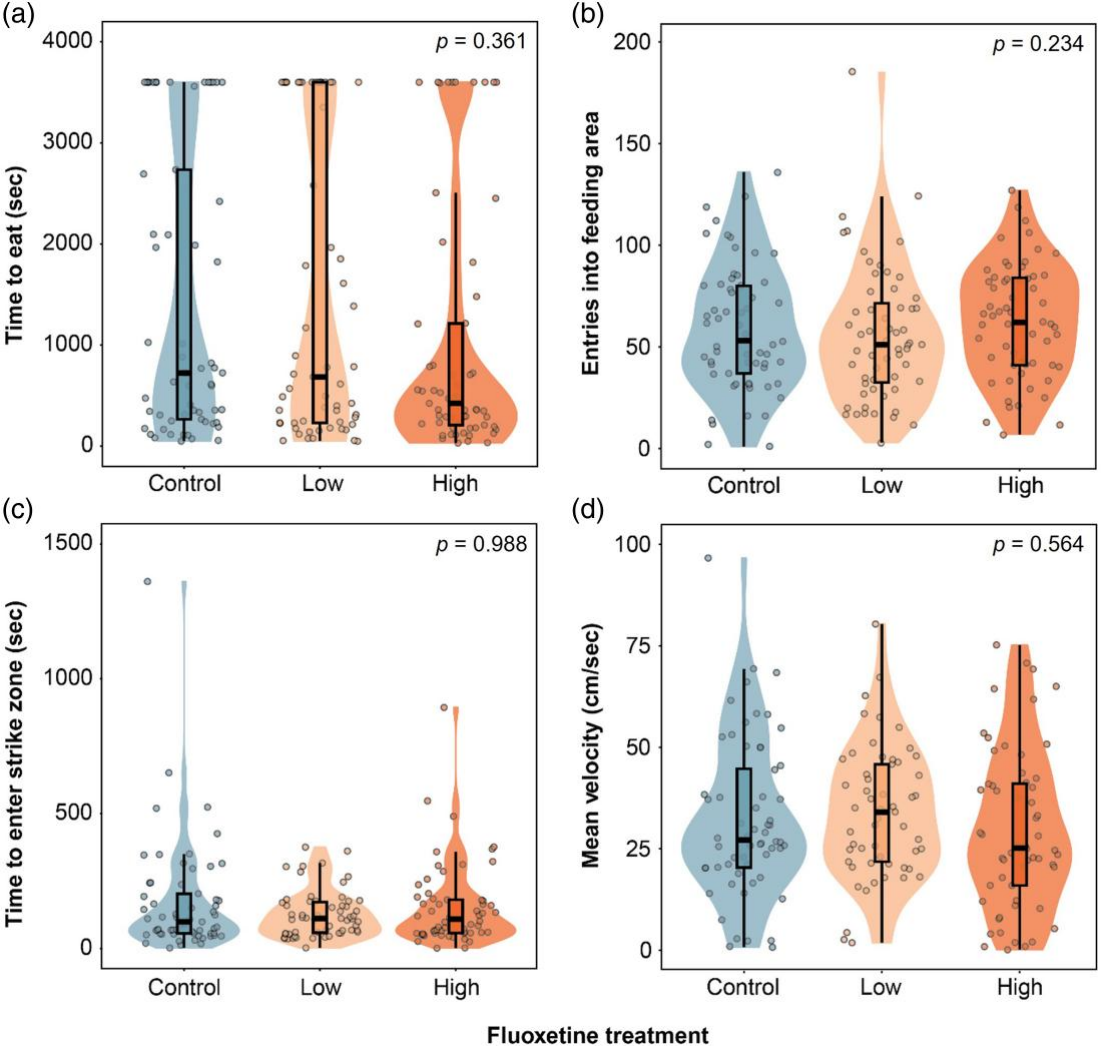
Boxplots and violin plots showing (a) time to consume a food item (s), (b) total number of entries into the feeding area, (c) time to enter the predator strike zone (s), and (d) swimming activity (mean velocity, cm/s), plotted for unexposed (*n* = 61), low-fluoxetine (*n* = 59), and high-fluoxetine (*n* = 61) female guppies, for predator absent and present treatments combined. Boxplots show the 25th, 50th (median), and 75th percentiles. The colored area surrounding the boxplot (violin plot) shows the probability density at different values smoothed by a kernel density estimator, while the points are the raw data (randomly spaced along the *x*-axis).

We found no significant main effect of fluoxetine treatment (*χ*^2^ = 2.037, df = 2, *P* = 0.361; Fig. 2a), predator treatment (*χ*^2^ = 0.520, df = 1, *P* = 0.471; Fig. S3), or water temperature (*χ*^2^ = 1.924, df = 1, *P* = 0.165) on the likelihood of guppies to consume a prey item. We did, however, detect an effect of experimental day, whereby guppies that were tested later in the experimental period were more likely to consume a food item (*χ*^2^ = 12.610, df = 1, *P* < 0.001; Fig. S4). Similarly, we found no significant effect of fluoxetine treatment on the number of entries into the foraging area (*χ*^2^ = 2.905, df = 2, *P* = 0.234; Fig. 2b). However, there was a significant effect of predator treatment (*χ*^2^ = 19.687, df = 1, *P* < 0.001). Specifically, guppies entered the foraging area more frequently when the predator was present compared with when it was absent (Fig. S3). Additionally, fish that were tested later in the experimental period entered the foraging area more frequently than those that were tested earlier (*χ*^2^ = 7.911, df = 1, *P* = 0.005; Fig. S5). There was no significant main effect of fluoxetine treatment on time taken to enter the predator strike zone (*F* = 0.012, df = 2, *P* = 0.988; Fig. 2c). Predator treatment had a significant effect on time to enter the strike zone, whereby fish entered the strike zone sooner when the predator was present compared with absent (*F* = 9.488, df = 1, *P* = 0.002; Fig. S3). Finally, regarding swimming activity, we found no effect of fluoxetine exposure (*F* = 0.638, df = 2, *P* = 0.564; Fig. 2d) or predator treatment (*F* < 0.001, df = 1, *P* = 0.993; Fig. S3) on mean velocity. However, experimental day had a significant effect on mean velocity, whereby fish that were tested later in the experimental period were more active than those that were tested earlier (*F* = 5.456, df = 1, *P* = 0.021; Fig. S6). Refer to Tables S5 and S6 for mean behavioral responses calculated for both fluoxetine treatments and predator treatments, respectively.

### Metabolic rate

**Figure 3:**
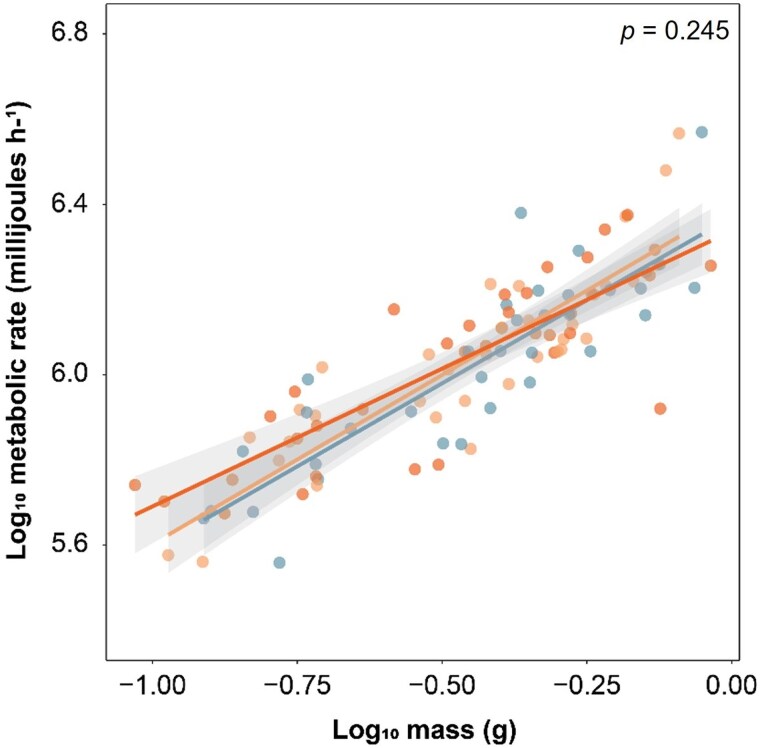
Scatterplot showing the predicted line of best fit (±SE) for the relationship between log10 mass (g) and log10 metabolic rate (millijoules h−1) plotted for unexposed (blue; *n* = 35), low-fluoxetine (light orange; *n* = 34), and high-fluoxetine (dark orange; *n* = 36) female guppies, with the *P*-value representing the main effect of fluoxetine treatment.

There was no significant main effect of fluoxetine treatment (*F* = 0.566, df = 2, *P* = 0.245; Fig. 3) or time of day (*F* = 1.257, df = 1, *P* = 0.265), and no significant interaction between body mass and fluoxetine exposure (*F* = 0.901, df = 2, *P* = 0.410) on metabolic rate in female guppies. However, there was a significant main effect of body mass, whereby females of a higher mass had, on average, higher metabolic rates (estimate ± SE = 0.908 ± 0.089 g, *F* = 103.776, df = 1, *P* < 0.001). Refer to Table S7 for mean metabolic rate values.

### Morphology

**Figure 4:**
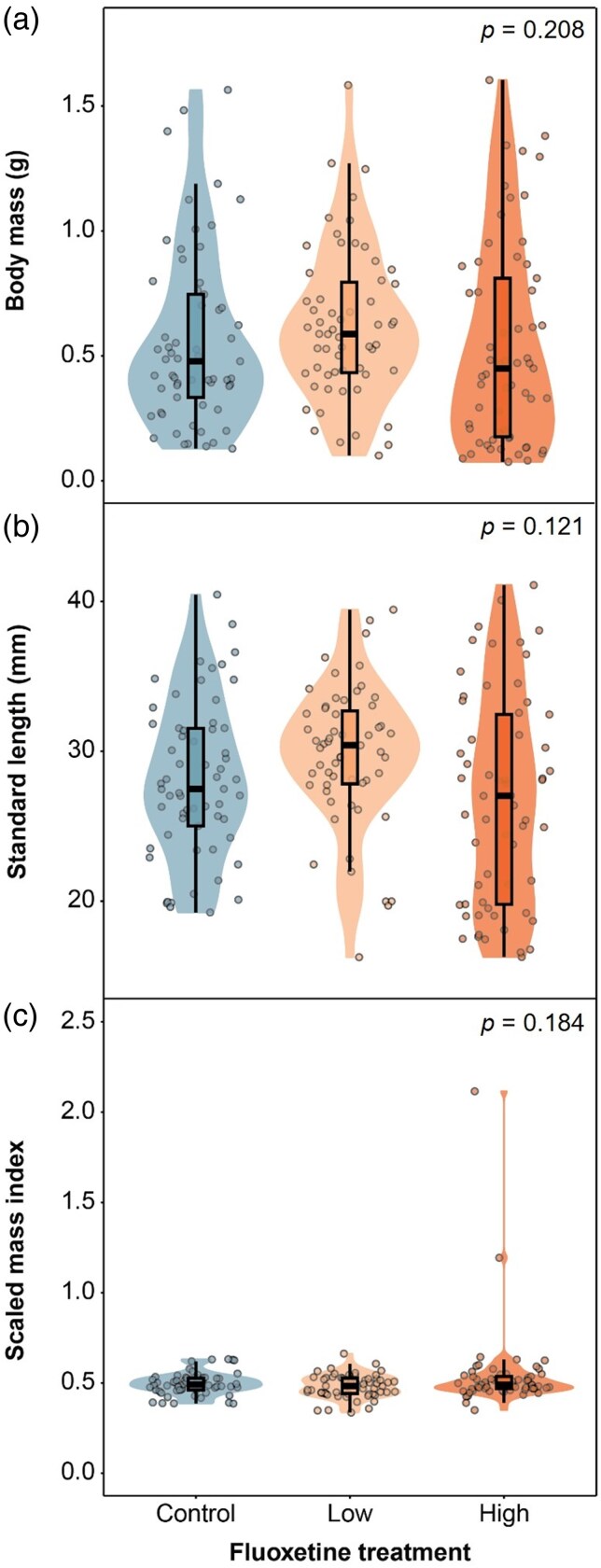
Boxplots and violin plots showing (a) body mass (g), (b) standard length (mm), and (c) scaled mass index, plotted for unexposed (*n* = 61), low-fluoxetine (*n* = 59), and high-fluoxetine (*n* = 61) female guppies. Boxplots show the 25th, 50th (median), and 75th percentiles. The colored area surrounding the boxplot (violin plot) shows the probability density at different values smoothed by a kernel density estimator, while the points are the raw data (randomly spaced along the *x*-axis).

We found no significant effect of fluoxetine exposure on body mass (*F* = 2.141, df = 2, *P* = 0.208; Fig. 4a), standard length (*F* = 3.238, df = 2, *P* = 0.121; Fig. 4b), or body condition (*F* = 2.834, df = 2, *P* = 0.184; Fig. 4c) in female guppies. Refer to Table S8 for mean morphological values.

## Discussion

We examined whether 8-mo exposure to the common pharmaceutical pollutant fluoxetine affected foraging and antipredator behavior, metabolic rate, and morphology in female guppies. Importantly, this study was conducted under ecologically relevant conditions in a semi-natural mesocosm system using environmentally realistic exposure concentrations (30 and 292 ng L^−1^). Additionally, we assessed foraging behavior under the perceived threat of predation. We found no evidence for an effect of fluoxetine on fish behavior under predation risk. Moreover, fluoxetine exposure had no significant effect on metabolic rate or morphology.

### Foraging under the perceived threat of predation

Acute fluoxetine-exposure studies have generally reported a reduction in antipredator behaviors in fish (eg Painter et al. 2009; Pelli and Connaughton 2015; Martin et al. 2017; but see Saaristo et al. 2017; Fursdon et al. 2019). If the impacts of chronic fluoxetine exposure reflected those of acute exposure studies, we would have expected to observe an increase in activity and foraging behavior in fluoxetine-exposed guppies in the presence of a predator—demonstrating a reduced behavioral response to the threat of predation. However, we did not detect any differences in antipredator behavior between exposed and unexposed female guppies. This result aligns with previous work in male guppies from the same mesocosm population (Fergusson et al. 2024). Despite behavior being studied under different ecological contexts (ie the presence of a foraging opportunity under predation risk), both studies found no effect on behavior under the same exposure conditions. Moreover, this finding is also consistent with previous long-term exposure studies in guppies (Tan et al. 2020; Polverino et al. 2022), and other species such as aquatic snails (*Physa acuta*; Henry et al. 2022) and daphnids (*Daphnia magna*; Heyland et al. 2020), where no clear effects of fluoxetine on behavior were detected (but see Polverino et al. 2021; Thoré et al. 2021b). One possible explanation for this is that the vast majority of previous studies have measured the impacts of fluoxetine exposure on antipredator behavior in isolation, not accounting for trade-offs with other ecologically important behaviors such as foraging, thereby making direct comparisons with our study difficult. Further, the findings of the few existing studies that have investigated behavior in a predation risk context do not align with the present study. For example, Thoré et al. (2019) reported alterations to antipredator behavior in turquoise killifish (*Nothobranchius furzeri*) in a foraging context, although such findings were only reported at fluoxetine concentrations an order of magnitude higher than those typically detected in aquatic environments (ie 5 μg L^−1^). At environmentally realistic concentrations, however, Peters et al. (2017) found that fluoxetine-exposed shore crabs (*Hemigrapsus oregonensis*) increased foraging and locomotor behavior in the presence of predators. Another potential reason for the discrepancy between our findings and those of previous studies is that behavioral responses to fluoxetine may vary with exposure duration. For instance, fluoxetine's mechanism of action has been linked to temporal changes in serotonergic signaling (Stewart et al. 2014), which may contribute to the differences observed between short- and long-term exposure studies. However, as our study did not include a direct comparison of exposure durations, this remains to be fully understood. Continued research into the long-term effects of pharmaceutical exposure in wildlife will therefore be highly beneficial, as the mechanisms driving variation in behavioral responses across exposure durations are not yet clear.

Although we found no effect of fluoxetine exposure on foraging or antipredator behavior in the presence of a predator, we did observe a change in guppy behavior between predator treatments (predator present versus empty compartment), irrespective of fluoxetine treatment. More specifically, both fluoxetine exposed and unexposed guppies entered the predator strike zone sooner (predator present mean± SE = 105.21 ± 9.26 s; predator absent mean ± SD = 197.10 ± 20.78 s), as well as entered the foraging area more frequently (predator present mean count ± SD = 69.04 ± 3.21; predator absent mean count ± SD= 49.40 ± 2.92), when the predator was present compared with when the predator was absent (Table S6). The decrease in time taken to enter the strike zone is not surprising as it aligns with predator inspection behavior, whereby guppies closely approach a potential predator to evaluate the level of threat that may be posed (Pitcher 1991; Godin and Davis 1995; Fishman 1999). This result is also consistent with previous studies that report changes in guppy behavior in response to predation threat (Fursdon et al. 2019; Tan et al. 2020; Mason et al. 2021). However, we note that the increased number of entries into the foraging area contrasts with results previously observed for predator-avoidance behavior in guppies (Fergusson et al. 2024). Here, it is possible that female guppies, in contrast to the male guppies tested in Fergusson et al. (2024), did not perceive the spangled perch as representing a substantial threat. However, it is important to note that between studies, behavior was measured under different ecological contexts. In the present study, guppies were provided with a foraging opportunity in the presence of a predator, whereas no such context was present in the previous work. In this regard, foraging motivation may have influenced the guppies’ response to predation risk. When presented with a decision between foraging and predation risk, the cost of a foraging opportunity may have been perceived as greater, and individuals may therefore be more willing to take risks to forage despite the presence of a predator. Finally, although the spangled perch were not exposed to fluoxetine in this study, we recognize the potential for both predator and prey species to be exposed to pollutants in the wild. Future studies may, therefore, examine the potential interactive effects of exposure to pharmaceutical contaminants in both predator and prey species, as exposure may influence species interactions (Bose et al. 2022).

### Standard metabolic rate

We did not detect a significant effect of fluoxetine exposure on metabolic rate in female guppies, which was contrary to our hypothesis. We expected to observe a change in metabolic rate because fluoxetine can reduce cortisol levels (Miranda et al. 2023) which can, in turn, influence metabolic rate (Mommsen et al. 1999). The lack of an observed effect on metabolic rate is consistent with that of male guppies under the same exposure conditions (Fergusson et al. 2024). By contrast, Tan et al. (2020) found a decrease in metabolic rate in female guppies exposed to 38 ng L^−1^ of fluoxetine. Although the present study and that of Tan et al. (2020) both investigated impacts of fluoxetine exposure on female guppies, a key difference in experimental design between these experiments was exposure duration. In our study, guppies were exposed to fluoxetine for 8 mo, whereas Tan et al. (2020) exposed guppies for 15 mo. Indeed, transgenerational studies (ie exposed parents, unexposed offspring) have demonstrated that effects of SSRI exposure can differ over successive generations. For instance, reduced cortisol levels and exploratory behavior (Vera-Chang et al. 2018), increased size (Heyland et al. 2020), and altered fecundity (Minguez et al. 2015; Heyland et al. 2020) have been observed in the unexposed offspring of parents exposed to an SSRI. In addition to differences in exposure duration, we also measured metabolic rate under nonstressful conditions. Previous research has shown that oxygen uptake in fish can increase in the presence of a predator (Palacios et al. 2016; Tan et al. 2020). Given that fish were potentially exposed to higher levels of stress in behavioral trials, it could therefore be beneficial to further explore how fluoxetine may impact metabolic rate under similar conditions.

Differences in respirometry methodologies may also explain the lack of any observed effect of fluoxetine exposure on metabolic rate. For instance, using a closed respirometry system with no mixing device could have affected precision in comparison to intermittent flow respirometry (Clark et al. 2013). However, we used a relatively long measurement period, ensuring a robust calculation of oxygen concentration over time (Svendsen et al. 2016). Additionally, a high respirometer-to-fish volume ratio ensured that fish did not reach a critical oxygen pressure within the vials. Finally, body mass was found to be a reliable predictor of metabolic rate (*R*^2^ = 0.73) despite no effect of fluoxetine exposure, given the significant allometric scaling relationship consistent with previous work on other taxa (White and Kearney 2014; White et al. 2019).

### Morphology

We did not detect an effect of fluoxetine exposure on body mass, standard length, or body condition in female guppies. This finding is generally not concordant with previous studies investigating the impacts of fluoxetine exposure on morphology in fish, where alterations in these traits have been reported (eg Pelli and Connaughton 2015; Bertram et al. 2018; Wiles et al. 2020; but see Fursdon et al. 2019; Fergusson et al. 2024). As discussed above regarding metabolic rate, exposure duration may, in part, explain the difference in results. More specifically, short-term fluoxetine exposure studies have reported reduced weight and standard length in guppies (Pelli and Connaughton 2015), and reduced body condition in eastern mosquitofish (*Gambusia holbrooki*; Bertram et al. 2018). This can be attributed to a decrease in food consumption (Bertram et al. 2018), as previously observed in fluoxetine-exposed fish (reviewed in Correia et al. 2023). Overall, this supports the idea that fluoxetine, and other SSRIs more broadly, may elicit duration-dependent (Stewart et al. 2014) or generation-specific effects (Minguez et al. 2015; Vera-Chang et al. 2018; Heyland et al. 2020; Thoré et al. 2021b).

In light of the absence of detectable effects across all of the behavioral, physiological, and morphological traits measured in our study, we note that the interpretation of such results should be sensitive to statistical power. As post hoc power analyses are not considered informative (Hoenig and Heisey 2001; Nakagawa and Foster 2004), we instead focus on the magnitude, direction, and consistency of estimated effects across models (Nakagawa and Cuthill 2007). In the present study, estimated effects of fluoxetine were small in magnitude, and showed no consistent directional pattern. By contrast, we detected clear effects of biologically relevant predictors including predator treatment, experimental day, and body mass (in the case of metabolic rate). Importantly, our sample sizes are comparable to, and in some cases exceed, those used in previous work assessing the ecological impacts of fluoxetine exposure, where clear effects on behavior, physiology, and morphology have been reported (eg Peters et al. 2017; Tan et al. 2020; Wiles et al. 2020; Thoré et al. 2021b). Taken together, the absence of detectable effects of fluoxetine in our study, alongside consistently small and nondirectional effect size estimates, suggests that any potential effects are likely to be biologically small rather than obscured by low statistical power.

## Conclusion

In summary, we found no evidence for an effect of 8-mo fluoxetine exposure under semi-natural conditions on foraging and antipredator behavior, metabolic rate, or morphology (mass, standard length, scaled mass index) in female guppies. Our results contribute to growing evidence that the effects of fluoxetine exposure may be duration- or generation-dependent. While efforts to understand the long-term consequences of fluoxetine exposure are increasing, comparatively few studies have conducted environmentally realistic exposures beyond an ∼1-mo period. This underscores the need for continued research under extended time periods (eg over multiple generations) to not only understand the ecological impacts of pharmaceutical contaminants, but also the mechanism(s) that may underpin such effects.

## Supplementary Material

arag080_Supplementary_Data

## Data Availability

Analyses reported in this article can be reproduced using the data provided by Fergusson et al. (2026).
