# Supplementary material for: No effect of a widespread pharmaceutical pollutant on behavior, physiology, or morphology in guppies
Source: Behav Ecol. 2026 Jul 14;37(5):arag080. doi: 10.1093/beheco/arag080 (PMC13448303; doi:10.1093/beheco/arag080)
Supplement: Supplementary file 1 [file arag080_supplementary_data.zip › Supplementary_File_1_Supplementary_Material.pdf]

Supplementary Information for

No effect of a widespread pharmaceutical pollutant on behavior, physiology, or morphology in guppies

Kate N. Fergusson[1,*], Stephanie L. Hannington[1], Jack A. Brand[1,2,3], James L. Tanner[1], Amanda K. Pettersen[1,4], Celine T. Goulet[1], Josefin Sundin[5], Minna Saaristo[1,6], Michael G. Bertram[1,3,7,†], Bob B.M. Wong[1,†], Jake M. Martin[1,3,8,†]

[1] *School of Biological Sciences, Monash University, 25 Rainforest Walk, Clayton, Victoria 3800, Australia*

[2] *Institute of Zoology, Zoological Society of London, Outer Circle, Regent's Park, London NW1 4RY, United Kingdom*

[3] *Department of Wildlife, Fish, and Environmental Studies, Swedish University of Agricultural Sciences, Skogsmarksgränd 17 Umeå 907 36, Sweden*

[4] *School of Life and Environmental Sciences, The University of Sydney, Science Road, Camperdown, New South Wales 2050, Australia*

[5] *Department of Aquatic Resources, Swedish University of Agricultural Sciences, Stångholmsvägen 2, SE-17893, Drottningholm, Sweden*

[6] *Environment Protection Authority Victoria, EPA Science, Ernest Jones Drive, Macleod, Victoria 3085, Australia*

[7] *Department of Zoology, Stockholm University, Svante Arrhenius väg 18b, Stockholm 114 18, Sweden*

[8] *School of Life and Environmental Sciences, Deakin University, 75 Pigdons Road, Waurn Ponds, Victoria 3216, Australia*

* Corresponding author: School of Biological Sciences, Monash University, 25 Rainforest Walk, Clayton, Victoria 3800, Australia. Email: Kate.Fergusson@monash.edu

[†] *Contributed equally as senior authors*

1. Supplementary tables

Table S1. Summary table for average (± standard deviation, SD) water temperature (°C) and pH for each mesocosm tank during the 8-month exposure period. *n* = the number of times temperature and pH were measured.

Tank	Temperature (°C)			pH		
	n	**Mean (±SD)**	**SE**	***n***	**Mean (±SD)**	**SE**
C1	43	23.90 ± 0.96	0.15	43	6.95 ± 0.90	0.14
C2	44	23.19 ± 0.77	0.12	43	7.31 ± 0.73	0.11
C3	44	23.22 ± 0.86	0.13	43	7.37 ± 0.66	0.10
C4	44	23.33 ± 0.53	0.08	43	6.77 ± 0.74	0.11
L1	42	22.14 ± 1.00	0.15	42	7.28 ± 0.70	0.11
L2	44	22.97 ± 0.95	0.14	43	7.11 ± 0.29	0.05
L3	42	23.57 ± 0.44	0.07	43	7.23 ± 0.42	0.06
L4	44	22.56 ± 0.82	0.12	43	7.07 ± 0.29	0.04
H1	44	23.63 ± 0.69	0.10	43	7.03 ± 0.78	0.12
H2	42	22.63 ± 1.16	0.18	43	7.56 ± 0.53	0.08
H3	44	23.38 ± 0.62	0.09	43	7.16 ± 0.86	0.13
H4	43	23.57 ± 0.37	0.06	43	6.94 ± 0.65	0.10

Table S2. Detailed list of methodological information for reporting methods of aquatic respirometry, following the guidelines of Killen *et al.* (2021).

Required information (Killen *et al.* 2021)	Answers
**Equipment**	
Provide body mass of animals at time of respirometry	Mean ± SE = 405.64 ± 20.04 mg (n = 105); see data file for individual mass
Provide volume of respirometer	100 mL
Provide volume of tubing in mixing circuit	NA
Provide ratio of animal body mass to volume of respirometer (plus associated tubing in mixing circuit)	405.64:100
Provide material of respirometer (e.g. glass, acrylic, etc.)	Glass
Provide material of tubing	NA
Provide type of oxygen probe and data recording	24-channel PreSens sensor dish reader (Sensor Dish Reader, SDR2, PreSens Precision Sensing, Germany)
Provide sampling frequency of water oxygen concentration	60 s
Describe how chamber mixing was achieved	By animal movement, no mixing device was used
Describe placement of oxygen probe (in recirculation circuit or directly in chamber)	NA; non-consumptive oxygen sensor spot on the base of each vial
Provide flow rate during flushing and recirculation, or confirm that chamber returned to normoxia during flushing	NA
Declare whether volume of tubing in mixing circuit was included in calculations of oxygen uptake	NA

Provide timing of flush/closed cycles	NA
Provide wait (delay) time excluded from closed measurement cycles	The first 30 min was excluded from analysis to allow the fish to acclimate
Describe frequency and method of probe calibration (for both 0 and 100% calibrations)	Sensor spots for each vial calibrated (0 and 100%) at the start of the experiment
Mention whether software temperature compensation was used during recording of water oxygen concentration	Software temperature was recorded along with oxygen concentration using PreSens v4 software
**Measurement conditions**	
Provide temperature during respirometry	24 °C
Describe how temperature was controlled	Ambient
Provide photoperiod during respirometry	All measurements were recorded in dark conditions
Provide minimum water oxygen level or concentration reached during closed phases	73% air saturation
Describe whether chambers were visually shielded from external disturbance	Yes. A cover was placed over the vials and the room lights were turned off to protect from external disturbance
Describe how many fish were measured during a given respirometry trial	Three fish were measured during each measurement period, using one of four glass vessels submerged in separate water baths
If multiple animals were measured simultaneously, describe whether they were able to see each other during measurements	Guppies were kept in dark conditions, such that they were not able to see each other
Provide duration of animal fasting before placement in respirometer	24 h
Provide duration of all trials combined (number of days to measure all animals in the study)	The total time to conduct all trials was 3 days

Provide acclimation time to the laboratory before respirometry measurements	Guppies were sourced from mesocosm population that were acclimated for 5 months before an 8-month exposure period
**Background respiration**	
Mention whether background respiration was measured and accounted for, and if so, how this was done	Yes. One control vial per group containing only water was used to account for background respiration rates
Specify method used to measure background respiration (e.g. parallel measures with empty respirometry chamber, measurements before and after for all chambers while empty, both)	Trials were run in groups of four, with each group containing three animals and one blank (i.e. empty vessel)
If background respiration was measured at beginning and end, describe how many slopes and for what duration	It was not. It was measured during each round
Describe how were changes in background respiration modelled over time (e.g. linear, exponential, parallel measures)	Same as the data for vials containing individuals (a single slope, with first 30 mins of recording discarded)
Provide level of background respiration (e.g. as a percentage of SMR)	4–15%
Describe method and frequency of system cleaning (e.g. system bleached between each trial, UV lamp)	Vials were cleaned with 10% ethanol, as per the manufacturer's instructions, after each measurement run
**Standard or routine metabolic rate**	
Provide acclimation time after transfer to chamber, or alternatively, time to reach beginning of metabolic rate measurements after introduction to chamber	30 min
Provide duration over which metabolic rate was estimated	2.5 h

Table S3. Summary of the global and selected generalized linear mixed-effects models (GLMMs) and linear mixed-effects models (LME) used for each response variable in the behavioral assay. Where the inclusion of fixed effects in the global model did not improve model fit, as tested by Akaike information criterion comparisons, they were removed from the model.

Response	Model type	Global model	Selected model
Time taken to consume a food item (sec)	Binomial GLMM	**Fixed effects**<br>Fluoxetine:predator<br>Fluoxetine:mass<br>Fluoxetine<br>Predator<br>Mass<br>Experimental day<br>Temperature<br>Foraging side<br>**Random effects**<br>Mesocosm ID	**Fixed effects**<br>Fluoxetine<br>Predator<br>Experimental day<br>Temperature<br>**Random effects**<br>Mesocosm ID
Number of entries into the feeding area	Poisson GLMM	**Fixed effects**<br>Fluoxetine:predator<br>Fluoxetine:mass<br>Fluoxetine<br>Predator<br>Mass<br>Experimental day<br>Temperature<br>Foraging side<br>**Random effects**<br>Mesocosm ID<br>Fish ID	**Fixed effects**<br>Fluoxetine<br>Predator<br>Experimental day<br>**Random effects**<br>Mesocosm ID<br>Fish ID
Time to enter the strike zone (sec)	LME model (log transformed)	**Fixed effects**<br>Fluoxetine:predator<br>Fluoxetine:mass<br>Fluoxetine<br>Predator<br>Mass<br>Experimental day<br>Temperature<br>Foraging side<br>**Random effects**<br>Mesocosm ID	**Fixed effects**<br>Fluoxetine<br>Predator<br>**Random effects**<br>Mesocosm ID

Mean velocity (cm/sec)	LME model	**Fixed effects**<br>Fluoxetine:predator<br>Fluoxetine:mass<br>Fluoxetine<br>Predator<br>Mass<br>Experimental day<br>Temperature<br>Foraging side<br>**Random effects**<br>Mesocosm ID	**Fixed effects**<br>Fluoxetine<br>Predator<br>Experimental day<br>**Random effects**<br>Mesocosm ID

Table S4. Summary table of raw and mean measured fluoxetine concentrations (ng L^{-1}) for each mesocosm tank during the 8-month exposure period (April–December 2017). No fluoxetine was detected in the control tanks (all under the limit of detection; <2 ng L^{-1}).

Tank	Month									Mean (±SD)
	April	May	June	July	August	September	October	November	December	
C1	<2	<2	<2	<2	<2	<2	<2	<2	<2	NA
C2	<2	<2	<2	<2	<2	<2	<2	<2	<2	NA
C3	<2	<2	<2	<2	<2	<2	<2	<2	<2	NA
C4	<2	<2	<2	<2	<2	<2	<2	<2	<2	NA
L1	4.4	25.6	25	57.6	32	41	46.6	43.7	30.9	34.1 ± 15.4
L2	7.8	25.9	24	34.8	17	22	50.2	38.5	35.2	28.4 ± 12.7
L3	8	35.1	22	36.6	28	14	40.8	39.7	33.3	28.6 ± 11.6
L4	4.6	29.9	24	35.4	22	21	45.1	44.6	33.2	28.9 ± 12.7
H1	52.8	330	270	560	580	190	277.2	209.6	158.8	292 ± 176.5
H2	59.6	350	280	480	290	100	199.5	175.8	140.9	230.6 ± 132.6
H3	41.3	390	300	330	510	270	485.9	267.2	142.5	304.1 ± 150.5
H4	75.5	340	280	420	450	260	668.1	384	190.1	340.9 ± 169.8

Table S5. Mean (±SE) behavioral responses of female guppies in the unexposed (0 ng L^{-1}; *n* = 61), low-fluoxetine (30 ng L^{-1}; *n* = 59), and high-fluoxetine (292 ng L^{-1}; *n* = 61) groups, for predator absent and present treatments combined.

Response variable	Fluoxetine treatment		
	**Unexposed**	**Low**	**High**
Latency to eat (sec)	1432.63 ± 180.21	1495.67 ± 193.57	1036.89 ± 61.57
Entries into foraging area	59.21 ± 3.97	56.03 ± 4.33	62.80 ± 3.59
Time to enter strike zone (sec)	170.00 ± 26.67	127.46 ± 11.40	152.97 ± 151.68
Mean velocity (cm/sec)	32.20 ± 2.49	33.82 ± 2.12	29.48 ± 2.50

Table S6. Mean (±SE) behavioral responses of female guppies in the predator absent (n = 89) and predator present (n = 92) groups, pooled across fluoxetine treatments.

**Response variable**	**Predator treatment**	
	**Absent**	**Present**
Latency to eat (sec)	1337.56 ± 146.50	1302.63 ± 147.42
Entries into foraging area	49.40 ± 2.92	69.04 ± 3.21
Time to enter strike zone (sec)	197.10 ± 20.78	105.21 ± 9.26
Mean velocity (cm/sec)	31.76 ± 1.92	31.84 ± 1.97

Table S7. Mean (±SE) metabolic rate (mJ h^{-1}) of female guppies in the unexposed (0 ng L^{-1}; *n* = 35), low-fluoxetine (30 ng L^{-1}; *n* = 34), and high-fluoxetine (292 ng L^{-1}; *n* = 36) groups, for predator absent and present treatments combined.

**Response variable**	**Fluoxetine treatment**		
	**Unexposed**	**Low**	**High**
Metabolic rate (mJ h^{-1})	1203870 ± 110503.8	1238159 ± 118521.0	1188443 ± 85634.4

Table S8. Mean (±SE) morphological characteristics of female guppies in the unexposed (0 ng L^{-1}; $n = 61$), low-fluoxetine (30 ng L^{-1}; $n = 59$), and high-fluoxetine (292 ng L^{-1}; $n = 61$) groups, for predator absent and present treatments combined.

Response variable	Fluoxetine treatment		
	Unexposed	Low	High
Body mass (mg)	559.99 ± 44.33	623.62 ± 39.25	535.16 ± 51.44
Standard length (mm)	28.11 ± 0.65	29.74 ± 0.61	26.70 ± 0.89
Body condition	0.50 ± 0.0076	0.48 ± 0.0083	0.54 ± 0.030

2. Supplementary figures

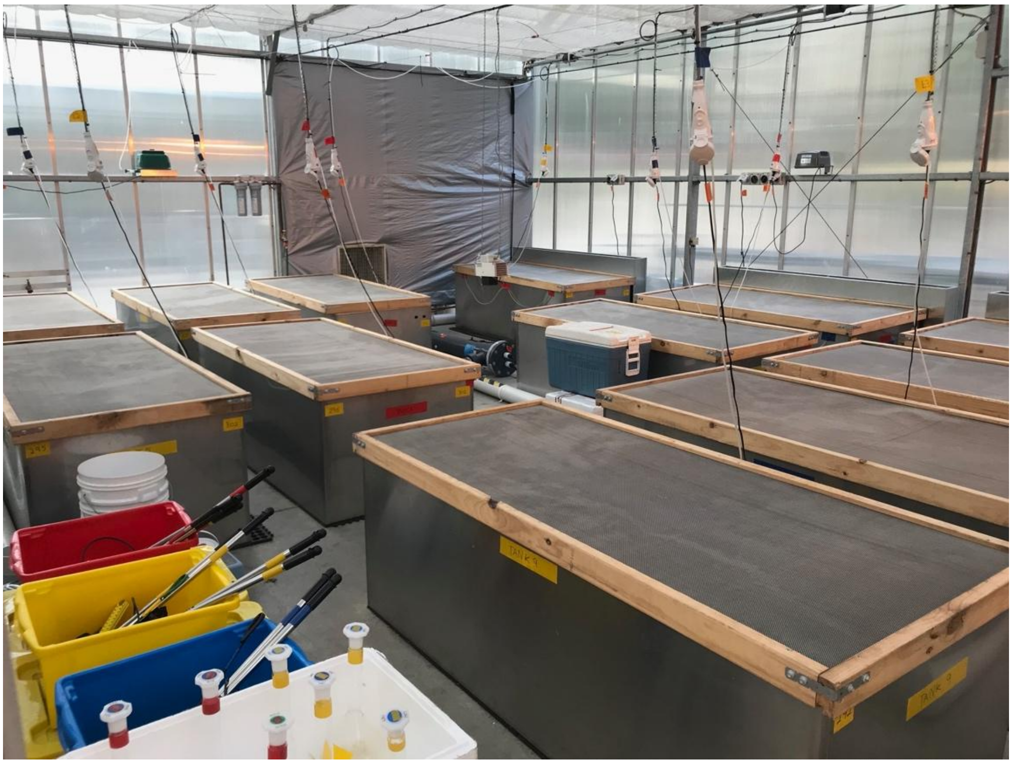

Figure S1. Photo of mesocosm tank set-up in the greenhouse facility at Monash University, Australia.

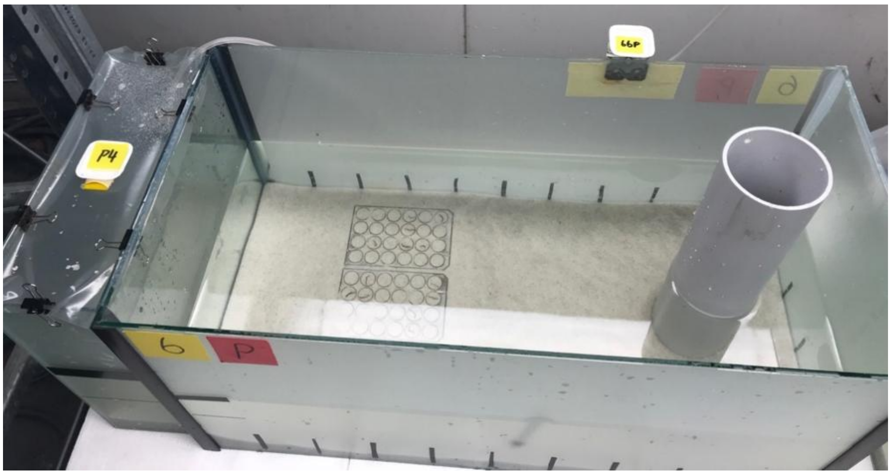

Figure S2. Photo of the observation tank (right compartment) containing a female guppy (in the photo, the guppy is in the opaque cylindrical chamber, which was lifted after the acclimation period). The foraging plate (a 72-well partially covered with sand, on top of which 20 bloodworms were placed) was placed 12 cm away from the predator compartment (left compartment) to ensure that the guppies had to approach the predator compartment to forage and, therefore, increase the perceived risk of predation to consume the food. The predator compartment contained a spangled perch (*Leiopotherapon unicolor*) in the predator treatment and was empty in the no-predator treatment. An ID number tag that did not reveal the treatment history of the guppies was placed in view of the camera on each observation tank to enable blind analysis of the behavioral trial videos. An ID number of the predator was placed in view of the camera on each predator compartment to enable including predator ID in the statistical analyses.

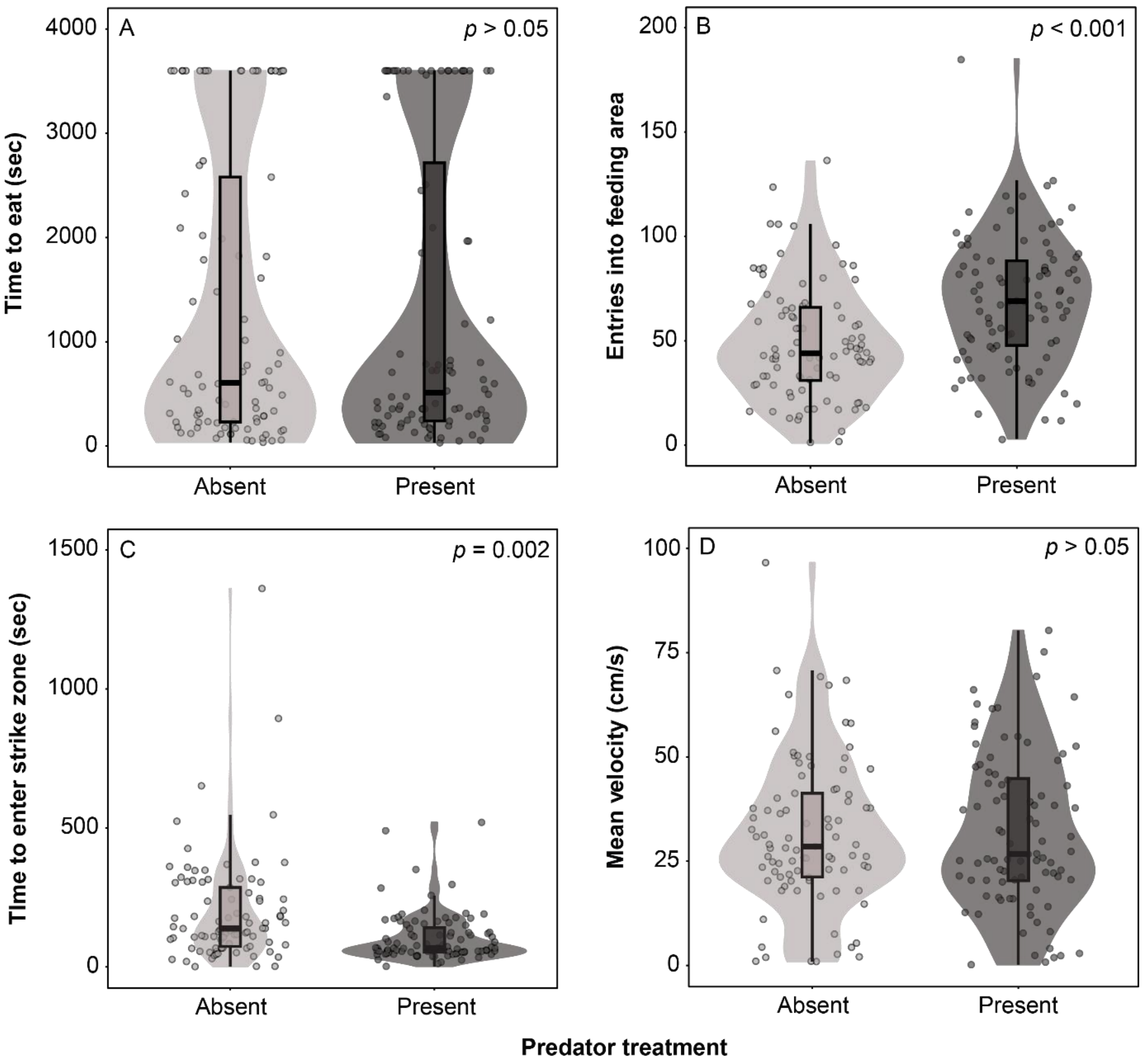

Figure S3. Boxplots and violin plots showing (A) time taken to consume a food item (sec), (B) total number of entries into the feeding area, (C) time taken to enter the predator strike zone (sec), and (D) mean velocity (cm/s), plotted for female guppies in the predator absent (light grey; *n* = 89) and predator present (dark grey; *n* = 92) groups, pooled across fluoxetine treatments. Boxplots show the 25th, 50th (median), and 75th percentiles. The colored area surrounding the boxplot (violin plot) shows the probability density at different values smoothed by a kernel density estimator, while the points are the raw data (randomly spaced along the x-axis).

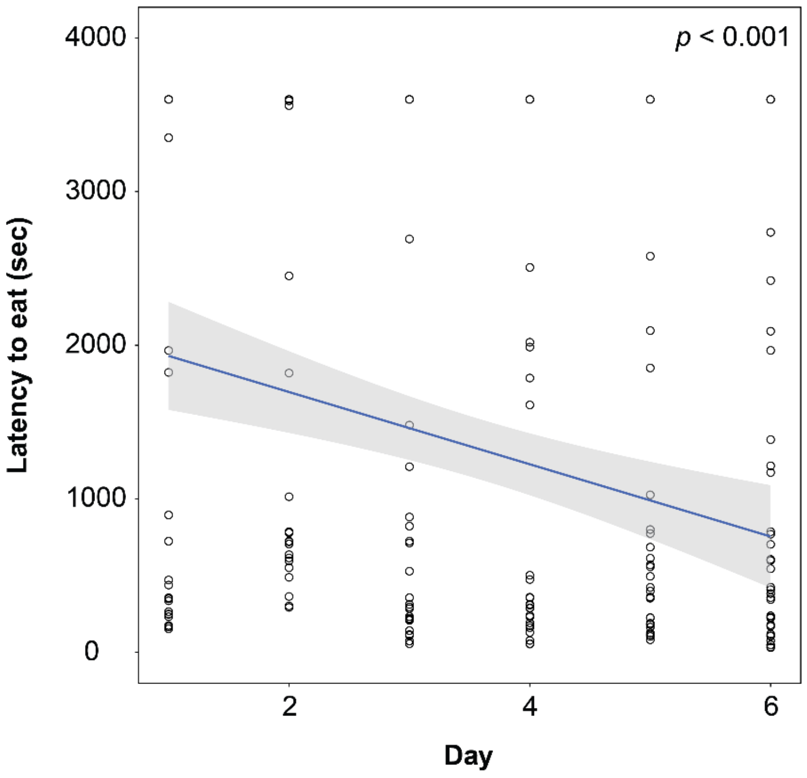

Figure S4. Scatterplot showing the predicted line of best fit (±SE) for the relationship between the time taken for guppies to first consume a food item (sec) and experimental day (1–6), pooled across fluoxetine treatments (n = 181). Foraging and antipredator behavior of female guppies were examined in the presence of a live predator, the spangled perch (*Leiopotherapon unicolor*) following an 8-month fluoxetine exposure period.

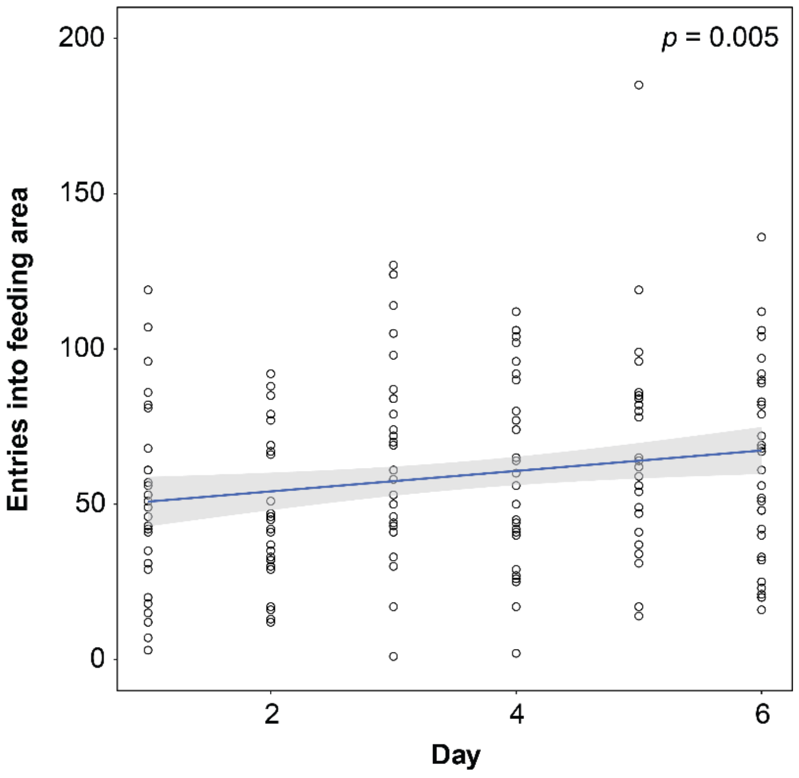

Figure S5. Scatterplot showing the predicted line of best fit (±SE) for the relationship between the number of entries into the feeding area and experimental day (1–6), pooled across fluoxetine treatments (*n* = 181). Foraging and antipredator behavior of female guppies were examined in the presence of a live predator, the spangled perch (*Leiopotherapon unicolor*) following an 8-month fluoxetine exposure period.

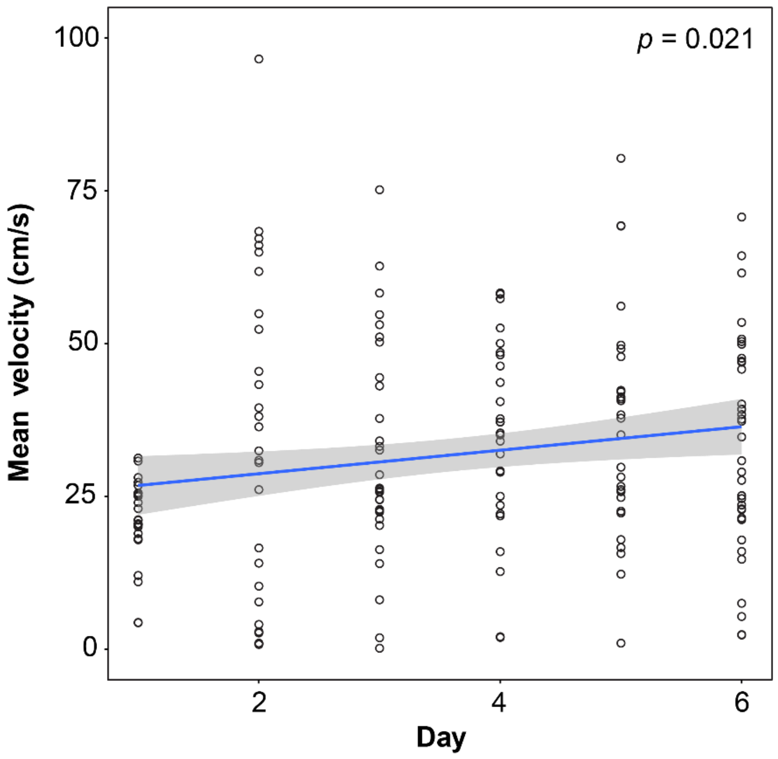

Figure S6. Scatterplot showing the predicted line of best fit (±SE) for the relationship between mean velocity (cm/s) and experimental day (1–6), pooled across fluoxetine treatments (n = 177). Foraging and antipredator behavior of female guppies were examined in the presence of a live predator, the spangled perch (*Leiopotherapon unicolor*) following an 8-month fluoxetine exposure period.
